# Loneliness, loneliness literacy, and change in loneliness during the COVID-19 pandemic among older adults: a cross-sectional study

**DOI:** 10.1186/s12877-022-03396-7

**Published:** 2022-08-26

**Authors:** Hui-Chuan Hsu, Shiau-Fang Chao

**Affiliations:** 1 School of Public Health, Taipei Medical University, No.250, Wuxing Street, 11031 Taipei, Taiwan, R.O.C.; 2 Research Center of Health Equity, College of Public Health, Taipei Medical University, Taipei, Taiwan, R.O.C.; 3 Department of Social Work, National Taiwan University, Taipei, Taiwan, R.O.C.

**Keywords:** Loneliness, Loneliness literacy, COVID-19, Older adults, Mental well-being, Social support, Coping strategy, Community

## Abstract

**Background:**

Loneliness has become a significant public health concern for older people. However, little is known about the association of loneliness, loneliness literacy, and changes in loneliness during the COVID-19 pandemic with mental well-being. The purpose of this study was to explore whether loneliness literacy is related to a lower risk of loneliness, increased loneliness during the COVID-19 pandemic, and improved mental well-being for community-based older adults.

**Methods:**

A telephone survey was conducted to collect data from older adults aged 65 years or older in Taiwan (*n* = 804). Loneliness, change in loneliness during COVID-19, and loneliness literacy were the main variables. Mental well-being was assessed by depressive symptoms and life satisfaction. Related factors included personal level (demographics, health conditions, health behaviors, and problem-focused/ emotion-focused coping strategies), interpersonal level (marital status, living arrangements, social support, social participation, leisure activities, and social interactions during COVID-19), and societal level (areas and regions) factors.

**Results:**

Four dimensions of loneliness literacy were identified by factor analysis: *self-efficacy, social support, socialization, and in-home support*. *Self-efficacy* and *in-home support were* related to lower loneliness. Lower *self-efficacy*, higher *social support*, and higher *socialization* were related to changes (increases) in loneliness during COVID-19. *In-home support* may prevent depressive symptoms, while *self-efficacy* was beneficial for better life satisfaction. In addition, emotion-focused coping may increase loneliness during COVID-19, while satisfaction with family support would be a protective factor against loneliness.

**Conclusion:**

Loneliness literacy is related to loneliness and increased loneliness during the COVID-19 pandemic. Building up an age-friendly community with embedded services/information and learning positive coping and mental resilience strategies are suggested.

**Supplementary Information:**

The online version contains supplementary material available at 10.1186/s12877-022-03396-7.

## Introduction

Loneliness has become a significant public health concern for older people, especially within high-income countries [1]. Over 20% of community-dwelling older adults in Taiwan report feeling lonely sometimes or usually [2]. Perceived loneliness can have detrimental effects on mental health and well-being, including an increased risk of depression [3] and reduced life satisfaction [4]. High levels of loneliness in later life can accelerate the degeneration of the nervous system, hippocampus and other brain regions that are related to emotional regulation and cognition. Therefore, loneliness has been identified as a predictor of accelerated cognitive decline and the emergence of dementia [1, 5].

To minimize the spread of COVID-19, Taiwan enforced restrictions on physical and social contact, ranging from recommendations to maintain physical distance and stay at home orders to lockdowns of businesses and communities from May to August 2021 [6]. Notably, the suspension of community-based services and programs have caused significant and enduring impacts on the daily life and social interaction of older adults residing in communities, making them particularly vulnerable to increased loneliness and mental health problems [7–10]. However, the loneliness of older individuals has been affected differently by the COVID-19 pandemic. The resource perspective assumes that the degree to which an individual can access resources directly determines his or her feelings of loneliness and indirectly influences mental health by changing his or her social relations or levels of participation in activities during the pandemic [11]. Specifically, high levels of accessibility to resources across multiple dimensions, ranging from individual and materials to non-material, have enabled older persons to better manage restrictions and to maintain a desired lifestyle during the pandemic [11]. Thus, older adults with more resources would be less likely to experience increased loneliness, as well as decreased mental health and well-being because of COVID-19.

As loneliness refers to a subjective emotional state reflecting a lack of satisfaction with social relationships or discrepancy between desired and achieved levels of social relations [3, 12], loneliness literacy represents one’s behavioral, cognitive and social skills to access loneliness-related information [13]. The Loneliness Literacy Scale (LLS) [13] was derived from behavioral change theory and consists of four constructs, including: (1) awareness of and willingness to use loneliness-related services (motivation); (2) self-perception of one’s ability to find information and manage daily living (self-efficacy); (3) the availability of support from family, friends, and neighbors and the importance of their opinions (perceived social support); and (4) beliefs of importance to stay active within one’s social context (subjective norm). The validity and reliability of the LLS, as well as its association with loneliness, have been confirmed in a sample of older adults in the Netherlands. To the best of our knowledge, the LLS has not been tested in older adult populations of different cultures. Additionally, how loneliness and loneliness literacy influence changes in loneliness during the COVID-19 pandemic and the mental well-being of community-dwelling older people remains unknown.

Guided by the resource perspective [14], several resources that may be supplementary to loneliness or change in loneliness during COVID-19 and have the potential to influence the mental well-being of older people were considered, including (1) personal factors, such as age, sex, education, financial satisfaction, health condition, physical function, physical activity, coping strategies, COVID-19 worry, and COVID-19 quarantine experience [1, 3, 8, 15–17]; (2) interpersonal factors, such as marital status, living arrangement, employment status, social support and social participation, internet use, pet ownership, and changes in social interaction due to COVID-19 [15, 18–22]; and (3) societal factors, such as the area of residence [18].

Due to the gap in existing knowledge of this topic, the study aimed to, first, evaluate the extent to which loneliness literacy is related to loneliness and increased loneliness during the COVID-19 pandemic, and second, to determine how loneliness, loneliness literacy, and increased loneliness during the COVID-19 pandemic are associated with the mental well-being of older adults. Mental well-being that has been recognized as a consequence of loneliness includes depressive symptoms [1, 3, 5] and life satisfaction [4]. This research advances current knowledge by comprehensively assessing the resources at different levels and various manifestations of loneliness, the emotional response of loneliness, cognitive and behavioral abilities to access loneliness-related information (loneliness literacy), changes in loneliness during COVID, and how these all relate to depressive symptoms and life satisfaction.

## Methods

### Data and sample

The survey data were collected by telephone interviews from November to December 2021. The participants experienced the COVID-19 lockdown from May to August 2021. The sample was drawn from six areas in Taiwan based on the region. The sample size for each region was based on the age and sex distribution of the population, and the sample was stratified into four layers (age 65–74 and age 75; males and females). The community-based sample of older participants was recruited from the computer-assisted survey telephone interview system. The inclusion criteria included community-based older adults who were 65 years or older, able to communicate and with no severe cognitive impairment. Those who did not have autonomy due to physical or psychological impairments were excluded. If there was more than one person in the household, only one person was interviewed. No proxy was allowed. There were a total of 804 complete cases.

Interviewers were trained in conducting interviews and following research ethics. Participants were informed of the research purpose and their permission was obtained before the interview was conducted. Approval for the study was granted by the Institutional Review Board before it was conducted.

### Measures

The questionnaire draft was designed based on the literature review. Two experts in gerontology reviewed the questionnaire draft to assure content and face validity. Twenty-four older adults of different ages, sexes, and living areas (rural or urban) were recruited for the pretest to test the construct validity and reliability and to modify the wording of the questions. The final version of the questionnaire was then modified.

#### Loneliness, change in loneliness, and loneliness literacy

Loneliness was measured by the 6-item revised UCLA loneliness scale-6 [23] by asking about the frequency of the following feelings, for example: how often do you feel that you lack companionship, feel alone, or are no longer close to anyone? Each item was scored from 1 to 4, ranging from never to always. The total score of the 6 items was defined as loneliness. The Cronbach’s alpha of the loneliness literacy scale was 0.781. The participants were also asked if they agreed that loneliness has positive effects, scored from 1 to 5 from strongly disagree to strongly agree. In addition, an open-ended question about when the participants would feel lonely was asked to understand the possible reasons or context for loneliness.

The change in loneliness due to COVID-19 was assessed by asking how the feeling of loneliness changed during COVID-19. Responses included: loneliness increased a great deal, increased a little, did not change, reduced a little, or reduced a great deal. The response was then categorized as “reduced or no change” vs. “increased”.

Loneliness literacy was modified from the Loneliness Literacy Scale (LLS) [13]. Based on the results of the pretest and the evaluation of the validity by two experts, only the 15 items from the scale were used (please see Supplement Table S1). The sample questions were as follows: able to attend activities alone, able to find information in daily life, family would help if you ask, friends would help you if you ask, you are aware of information regarding exercise programs, entertainment or courses, etc. The items were scored from 1 to 5, from strongly disagree to strongly agree. The Cronbach’s alpha of the loneliness literacy scale was 0.769, indicating high internal consistency. The factor analysis result of loneliness literacy is shown in Supplement Table S2. Four dimensions of loneliness literacy were extracted by factor analysis: *self-efficacy, social support, in-home support, and socialization*; the explained variance was 55.94%. Then, the four levels of literacy were calculated as the average score of the sum of the items for each dimension.

#### Mental well-being

Mental well-being included depressive symptoms and life satisfaction. Depressive symptoms were measured by the revised 10-item version of the Center for Epidemiological Studies Depression scale [24]. The sample items were as follows: I felt depressed; I felt that everything I did was an effort; I could not get going, etc. Each item was scored from 0 to 3; the total score ranged from 0 to 30. The CESD-10 scale was validated and tested in previous research using T transformation on nation-representative data in Taiwan [25]; a score of 8 or more was defined as depressive. The Cronbach’s alpha of the CESD-19 in this study was 0.59, indicating a slightly lower internal consistency. Life satisfaction was measured by one question asking if participants felt satisfied about their life, and the score was from 1 to 5 and then categorized into two groups (unsatisfied/fair and satisfied).

#### Personal factors

Individual factors included health conditions, health behaviors, coping strategies, and demographics. Physical health conditions included self-rated health and physical function. Self-rated health was scored from 1 to 5, indicating poor to excellent health. Physical function was measured by three kinds of activities: activities of daily living (including dressing, transferring, walking indoors, using the toilet, taking a bath, and eating), indoor instrumental activities of daily living (cooking or preparing meals, laundry, doing housework), and outdoor instrumental activities of daily living (walking outdoors, going out by transportation alone, and shopping for groceries). Each item asked if there was any difficulty in any of the activities of that kind (based on the most difficult one). Difficulty was scored from 0 to 3 to represent not difficult, slightly difficult, very difficult, and unable to do. The total score of the three kinds of physical function difficulty ranged from 0 to 9. Regarding vision and hearing, participants were asked if there was any impairment (yes/no).

Health behaviors included smoking (never, quit, current smoking), physical activity or exercise (frequency in a week and minutes every time), and drinking frequency (scored 0 to 4, indicating never to every day). Physical activity was measured in minutes per week, and regular physical activity was defined as 150 min or more (yes or no).

Coping strategies were measured according to the coping strategies of older Taiwanese individuals [26] by asking the frequency with which the participants engaged in coping behaviors when they were in distress. Each item was scored from 1 to 4, indicating never, seldom, sometimes, and always. According to the results of factor analysis, the coping strategies were categorized into two types: problem-focused coping (taking action to reduce worry/distress, doing something else to distract from distress, leaning to live with worry/distress, and praying or meditation to get comfort from religious beliefs) and emotion-focused coping (seeking out care from others, not believing that worry/distress has happened, continuously venting, and blaming yourself for letting it happen). The two kinds of coping strategy scores were the summed scores of the items of each kind.

Other personal demographic variables included age, sex, education (illiterate, elementary school, junior high school, senior high school, college or university, graduate and above), religious belief (yes/no), and financial satisfaction (scored 1–5).

#### Interpersonal factors

Interpersonal factors included marital status (having a spouse or not), living arrangement (living with others or alone), social support, social participation, leisure activities, and social interaction changes during COVID-19.

Social support was measured by social contact frequency and satisfaction with social support. The contact frequency of family and of friends/neighbors at least once per week was defined as high; otherwise, it was defined as low. Satisfaction of social support from family or from friends/neighbors was scored from 0 to 5. Social participation was defined as working (yes/no), taking care of family or children (yes/no), and participating in volunteer or social groups (yes/no). Leisure activities included physically active leisure (exercise, dancing, traveling) or sedentary leisure activity (such as reading, watching movies, painting, and calligraphy) (yes/no). Using the internet at least once per week was defined as being an active user. The participants were also asked if they had pets (yes/no).

The influences of COVID-19 were added to this survey, including whether social participation, leisure activity and interaction with relatives and friends changed during the COVID-19 pandemic (reduced a great deal, reduced a little, no change, increased a little, and increased a great deal); worry or fear because of COVID-19 (not at all, occasionally, usually, and always); and experience of COVID-19 quarantine (yes/no).

#### Societal factors

Societal factors were assessed by the place where the participants lived, including regions and rural/urban areas. Four regions of Taiwan were coded: North, Central, South, and East. The living area was also defined as rural or urban, which was defined according to the city development definition [27].

### Analysis

Descriptive analysis, bivariate analysis, factor analysis, linear regression analysis, and logistic regression analysis were conducted. Factor analysis was used to extract the components of loneliness literacy. Linear regression analysis was used to examine factors related to loneliness. The factors related to increased loneliness during COVID-19 and mental well-being were first analyzed by logistic regression.

## Results

Table 1 shows a description of the sample. The average age was 73.74 years old; males and females accounted for 45.6% and 54.4% of the participants, respectively. The average mean loneliness score was 8.01 (score 6 to 24). During COVID-19, 61.1% of the participants had reduced interactions with family or friends, and 26.4% reported change in loneliness increased. Most people did not worry or feel afraid due to COVID-19 (mean = 1.50, SE = 0.83). Only 0.9% had experienced COVID-19 quarantine. Bivariate analysis was conducted to examine all the related factors to loneliness and changes in loneliness; the significant factors were included in the following multivariate analysis.

**Table 1:** Description of the sample characteristics

Variables	Mean (SD) or %
**Demographics**	
Age	73.74 (6.11)
65–74 years old	63.8%
75 years old and above	36.2%
Sex	
Females	54.4%
Males	45.6%
Marital status	
No spouse	29.2%
Having spouse	70.8%
Education (0 ~ 5)	2.46 (1.34)
Religion belief	
No	14.6%
Yes	85.4%
Financial satisfaction (1 ~ 5)	3.61 (0.94)
Living arrangement	
Alone	11.6%
With others	88.4%
Regions	
North: Taipei, New Taipei, Keelung	31.3%
Middle-North: Taoyuna, Hsinchu, Miaolee	13.6%
Middle: Taichung, Changhwa, Nantou	18.2%
Middle-South: Yunlin, Chiayi, Tainan	20.5%
South: Kaohsiung, Pingtung	16.4%
East: Yilan, Hwalien, Taitung	5.0%
Residency	
Urban	70.7%
Rural	29.3%
**Health and health behaviors**	
Self-rated health (1 ~ 5)	3.32 (1.13)
Physical function difficulty (0 ~ 9)	0.56 (1.52)
Vision (clear %)	85.1%
Hearing (clear %)	92.9%
Regular physical activity	
No	36.9%
Yes	63.1%
Smoking	
Never	81.0%
Quitted	12.1%
Smoking	7.0%
Drinking frequency (0 ~ 4)	
Never	81.0%
Seldom	12.1%
Sometimes	7.0%
Always	0.0%
Depressive symptoms (0 ~ 30)	5.48 (5.53)
Yes (score > = 8)	27.3%
No (score < 8)	72.7%
Coping strategies	
Problem-focused coping	10.42 (3.03)
Emotion-focused coping	6.69 (2.32)
Life satisfaction (1 ~ 5)	4.10 (0.85)
Low or fair	12.3%
High	87.7%
**Social support, social participation, and leisure**	
Working (yes %)	11.4%
Family contacts (> = 1/week)	90.4%
Friends or neighbor contact (> = 1/week)	84.9%
Satisfaction of family support (0 ~ 5)	4.25 (0.85)
Satisfaction of friends/neighbors support (0 ~ 5)	3.86 (1.19)
Using Internet (at least once per week)	61.2%
Having pets (yes)	13.3%
Family care (care of family or children) (yes)	30.3%
Participating in volunteers or social groups (yes)	30.3%
Physically active leisure (yes)	28.0%
Sedentary leisure (yes	61.2%
**Loneliness**	
Loneliness total score (6 ~ 24)	8.01 (3.28)
Positive effect of loneliness (1 ~ 5)	3.00 (1.10)
Loneliness literacy total score	16.48 (10.74)
Dimension: *Self-efficacy*	3.84 (0.65)
Dimension: *Social Support*	4.82 (9.92)
Dimension: *In-home Support*	4.08 (3.76)
Dimension: *Socialization*	3.69 (0.74)
**Influence of COVID-19**	
Change of social participation or social interaction due to COVID-19	
Reduced a little or a lot	61.1%
No change	37.7%
Increased a little or a lot	1.1%
Change in loneliness due to COVID-19	
Reduced	1.0%
No change	72.6%
Increased	26.4%
Worried or afraid because of COVID-19 (1 ~ 4)	1.50 (0.83)
Experience in quarantine of COVID-19 (yes)	0.9%

Table 2 shows the factors related to loneliness using a linear regression model. The older participants who had lower financial satisfaction (B =  − 0.402, *p* < 0.01), had physical function difficulties (B = 0.315, *p* < 0.01), had lower *self-efficacy* (B =  − 0.638, *p* < 0.05), had lower *in-home support* (B =  − 0.184, *p* < 0.001), performed more problem-focused coping (B = 0.187, *p* < 0.001) and more emotion-focused coping (B = 0.171, *p* < 0.01), did not participate in social groups (B =  − 0.561, *p* < 0.05), and reported lower satisfaction of family support (B =  − 1.169, *p* < 0.001) were more likely to have a higher level of loneliness.

**Table 2:** Factors related to loneliness among the community-based older people by linear regression

Variables	B	SE	*P* value	95% C.I	
Constant	17.199	1.657	0.000	13.944	20.455
Region: Middle-North	0.282	0.371	0.447	-0.446	1.010
Region: Middle	-0.438	0.351	0.212	-1.126	0.251
Region: Middle-South	-0.378	0.367	0.304	-1.100	0.343
Region: South	0.399	0.366	0.277	-0.321	1.118
Region: East	-0.044	0.603	0.942	-1.227	1.140
Living area (rural)	-0.183	0.287	0.525	-0.747	0.381
Age (> = 75)	-0.422	0.265	0.112	-0.943	0.098
Sex (male)	-0.432	0.289	0.136	-1.000	0.137
Marital status (having spouse)	-0.515	0.310	0.097	-1.123	0.137
Education	0.093	0.105	0.376	-0.113	0.093
Financial satisfaction	-0.402	0.137	0.003	-0.670	-0.134
Living arrangement (alone)	0.239	0.431	0.579	-0.608	1.087
Self-rated health	-0.217	0.124	0.081	-0.461	0.027
Physical function difficulties	0.315	0.108	0.004	0.102	0.528
Smoking (yes)	0.406	0.560	0.469	-0.694	1.505
Drinking (yes)	0.333	0.401	0.407	-0.455	1.121
Physical activity (regular)	0.437	0.269	0.105	-0.092	0.966
Loneliness literacy: self-efficacy	-0.638	0.277	0.021	-1.181	-0.095
Loneliness literacy: social support	-0.146	0.163	0.370	-0.465	0.173
Loneliness literacy: socialization	0.117	0.185	0.528	-0.246	-1.113
Loneliness literacy: in-home support	-0.184	0.036	0.000	-0.254	-0.113
Problem-focused coping strategies	0.187	0.043	0.000	0.102	0.272
Emotion-focused coping strategies	0.171	0.054	0.002	0.066	0.277
Work (yes)	0.042	0.365	0.908	-0.675	0.759
Family care (yes)	-0.250	0.255	0.328	-0.752	0.251
Social group participation (yes)	-0.561	0.270	0.038	-1.091	-0.031
Family social contact (high)	-0.111	1.119	0.921	-2.308	2.086
Satisfaction of family support	-1.169	0.166	0.000	-1.494	-0.843
Satisfaction of friends support	-0.187	0.120	0.121	-0.424	0.049
Using Internet (yes)	-0.152	0.275	0.579	-0.692	0.387
Having pets (yes)	-0.041	0.346	0.905	-0.721	0.638
Physically active leisure (yes)	-0.222	0.275	0.420	-0.763	0.319
Sedentary leisure (yes)	0.323	0.280	0.249	-0.226	0.873
Model Fit	*R*^*2*^ = 0.329				

Note: Analysis by linear regression. The reference group: Loneliness during COVID-19 (no change or reduced), region (north), living area (urban), age (65–74), sex (female), marital status (no spouse), living arrangement (with others), smoking (no), drinking (no), physical activity (less than 150 min per week), work (no), family care (no), social group participation (no), family social contacts (low), using internet (less than once per week), having pet (no), physically active leisure (no), and sedentary leisure (no). Other variables were ordinal or continuous. The constant term was omitted in the table

Table 3 shows the factors related to increased loneliness during COVID-19 by a binary logistic regression model. The older participants who were male (OR = 1.878, *p* < 0.05), felt more loneliness (OR = 1.095, *p* < 0.05), had lower *self-efficacy* (OR = 0.458, *p* < 0.01), had more *social support* (OR = 1.595, *p* < 0.01), had more *socialization* (OR = 1.606, *p* < 0.01), performed more emotion-focused coping strategies (OR = 1.188, *p* < 0.001), had lower satisfaction with family support (OR = 0.651, *p* < 0.05), performed more physically active leisure activities (OR = 1.737), and were more worried about COVID-19 (OR = 2.095, *p* < 0.001) were more likely to experience increased loneliness during COVID-19.

**Table 3:** Association of increased loneliness during COVID-19 with related factors among the community-based older people by logistic regression (odds ratios)

Variables	Change in loneliness during COVID-19 (Increase)					
	B	SE	Exp(B) (OR)	*P* value	Lower 95% C.I. for Exp(B)	Upper 95% C.I. for Exp(B)
Constant	-2.211	1.594	0.110	0.165	--	--
Region: Middle-North	0.183	0.330	1.201	0.580	0.629	2.293
Region: Middle	0.303	0.314	1.353	0.335	0.731	2.505
Region: Middle-South	0.080	0.341	1.083	0.815	0.555	2.114
Region: South	-0.205	0.345	0.815	0.552	0.414	1.602
Region: East	-0.381	0.678	0.683	0.574	0.181	2.580
Living area (rural)	-0.432	0.271	0.649	0.111	0.381	1.104
Age (> = 75)	-0.210	0.251	0.811	0.402	0.496	1.326
Sex (male)	0.630	0.273	1.878	0.021	1.099	3.206
Marital status (having spouse)	-0.447	0.287	0.640	0.120	0.364	1.122
Education	0.016	0.098	1.016	0.867	0.839	1.231
Financial satisfaction	-0.057	0.125	0.944	0.646	0.740	1.207
Living arrangement (alone)	0.016	0.393	1.016	0.967	0.471	2.195
Self-rated health	0.013	0.115	1.013	0.910	0.808	1.269
Physical function difficulties	0.090	0.105	1.094	0.392	0.891	1.344
Smoking (yes)	-0.130	0.495	0.878	0.793	0.333	2.317
Drinking (yes)	0.203	0.347	1.226	0.558	0.621	2.418
Physical activity (regular)	-0.038	0.249	0.962	0.877	0.591	1.568
Loneliness	0.090	0.036	1.095	0.012	1.020	1.174
Loneliness literacy: *Self-efficacy*	-0.781	0.269	0.458	0.004	0.270	0.776
Loneliness literacy: *Social Support*	0.467	0.165	1.595	0.005	1.153	2.204
Loneliness literacy: *Socialization*	0.473	0.180	1.606	0.009	1.128	2.284
Loneliness literacy: *In-home Support*	-0.096	0.049	0.909	0.051	0.825	1.000
Problem-focused coping strategies	-0.008	0.042	0.992	0.851	0.914	1.077
Emotion-focused coping strategies	0.173	0.049	1.188	0.000	1.080	1.309
Work (yes)	0.112	0.343	1.118	0.744	0.571	2.191
Family care (yes)	-0.123	0.234	0.884	0.599	0.559	1.399
Social group participation (yes)	0.287	0.247	1.333	0.246	0.821	2.162
Family social contact (high)	-0.712	0.997	0.491	0.475	0.070	3.463
Satisfaction of family support	-0.429	0.166	0.651	0.010	0.471	0.902
Satisfaction of friends support	0.083	0.121	1.087	0.492	0.858	1.377
Using Internet (yes)	-0.051	0.256	0.950	0.842	0.576	1.570
Having pets (yes)	-0.326	0.341	0.722	0.340	0.370	1.408
Physically active leisure (yes)	0.552	0.263	1.737	0.036	1.038	2.908
Sedentary leisure (yes)	0.071	0.264	1.073	0.788	0.640	1.801
Worry about COVID-19	0.739	0.129	2.095	0.000	1.627	2.696
Model Fit: -2 log likelihood = 580.449, Chi-square = 148.490 (d.f. = 35), *p* < 0.001						

Note: Analysis by logistic regression. The reference group: Change in Loneliness during COVID-19 (no change or reduced), region (north), living area (urban), age (65–74), sex (female), marital status (no spouse), living arrangement (with others), smoking (no), drinking (no), physical activity (less than 150 min per week), work (no), family care (no), social group participation (no), family social contacts (low), using internet (less than once per week), having pet (no), physically active leisure (no), and sedentary leisure (no). Other variables were ordinal or continuous. The constant term was omitted in the table

Table 4 shows the associations of loneliness and other factors related to mental well-being by logistic regression analysis. Having depressive symptoms was significantly related to lower financial satisfaction (OR = 0.718, *p* < 0.05), poor self-rated health (OR = 0.682, *p* < 0.01), more loneliness (OR = 1.240, *p* < 0.001), having less *in-home support* (OR = 0.909, *p* < 0.05), performing more emotion-focused coping strategies (OR = 1.166, *p* < 0.01), not engaging in physically active leisure activities (OR = 0.521, *p* < 0.05), and having more worries about COVID-19 (OR = 1.363, *p* < 0.05). The participants who reported good life satisfaction were more likely to be female (OR = 0.273 for males, *p* < 0.05), had better financial satisfaction (OR = 3.361, *p* < 0.001), lived in the northern region as opposed to the central or central-southern regions, had better self-rated health (OR = 1.587, *p* < 0.05), reported lower loneliness (OR = 0.847, *p* < 0.05), and had higher *self-efficacy* (OR = 2.522, *p* < 0.05).

**Table 4:** Association of loneliness, loneliness literacy, change in loneliness and other factors with mental well-being among the community-based older people by logistic regression (odds ratios)

Variables	Depressive symptoms (*n* = 615)						Good life satisfaction (*n* = 623)					
	B	SE	Exp(B) (OR)	*P* value	Lower 95% C.I. for Exp(B)	Upper 95% C.I. for Exp(B)	B	SE	Exp(B) (OR)	*P* value	Lower 95% C.I. for Exp(B)	Upper 95% C.I. for Exp(B)
Age (> = 75 years old)	0.369	0.272	1.446	0.176	0.848	2.466	0.805	0.534	2.237	0.132	0.785	6.368
Sex (male)	-0.043	0.310	0.958	0.889	0.522	1.757	-1.300	0.552	0.273	0.019	0.092	0.805
Marital status (having spouse)	0.156	0.322	1.169	0.628	0.622	2.199	1.000	0.521	2.717	0.055	0.979	7.543
Education	-0.062	0.105	0.940	0.558	0.764	1.156	0.401	0.195	1.493	0.040	1.018	2.189
Financial satisfaction	-0.331	0.132	0.718	0.012	0.554	0.930	1.212	0.213	3.361	0.000	2.212	5.107
Living arrangement (alone)	0.169	0.433	1.184	0.697	0.507	2.766	1.074	0.753	2.927	0.154	0.669	12.806
Region: Middle-North	-0.179	0.390	0.836	0.647	0.389	1.797	-0.294	0.745	0.745	0.693	0.173	3.211
Region: Middle	0.170	0.347	1.185	0.625	0.600	2.339	-1.282	0.629	0.277	0.041	0.081	0.951
Region: Middle-South	0.172	0.388	1.188	0.658	0.555	2.542	-1.558	0.667	0.211	0.019	0.057	0.778
Region: South	-0.607	0.408	0.545	0.137	0.245	1.214	0.189	0.805	1.208	0.814	0.249	5.855
Region: East	0.509	0.624	1.664	0.414	0.490	5.654	-0.827	1.269	0.437	0.515	0.036	5.260
Living area (rural)	-0.283	0.305	0.753	0.353	0.415	1.369	0.159	0.496	1.173	0.748	0.444	3.097
Self-rated health	-0.383	0.122	0.682	0.002	0.536	0.867	0.462	0.227	1.587	0.042	1.016	2.477
Physical function difficulties	0.207	0.122	1.230	0.090	0.968	1.563	0.043	0.154	1.044	0.779	0.771	1.414
Smoking (yes)	0.472	0.578	1.603	0.414	0.516	4.973	-0.981	1.171	0.375	0.402	0.038	3.717
Drinking (yes)	-0.192	0.420	0.825	0.648	0.362	1.879	1.119	0.813	3.062	0.169	0.622	15.061
Physical activity (regular)	-0.285	0.271	0.752	0.293	0.442	1.279	0.828	0.492	2.288	0.092	0.873	5.997
Loneliness	0.215	0.043	1.240	0.000	1.140	1.349	-0.166	0.067	0.847	0.013	0.743	0.965
Loneliness literacy: *Self-efficacy*	-0.235	0.289	0.791	0.416	0.449	1.393	0.925	0.465	2.522	0.047	1.013	6.280
Loneliness literacy: *Social Support*	-0.141	0.171	0.869	0.409	0.622	1.213	-0.078	0.302	0.925	0.797	0.512	1.672
Loneliness literacy: *Socialization*	-0.326	0.192	0.722	0.089	0.495	1.051	-0.101	0.324	0.904	0.756	0.479	1.707
Loneliness literacy: *In-home Support*	-0.095	0.045	0.909	0.036	0.832	0.994	-0.049	0.050	0.953	0.329	0.864	1.050
Problem-focused coping	-0.032	0.049	0.969	0.517	0.881	1.066	0.026	0.084	1.026	0.757	0.871	1.210
Emotion-focused coping	0.154	0.055	1.166	0.005	1.046	1.300	-0.082	0.091	0.921	0.364	0.771	1.100
Work (yes)	0.063	0.396	1.065	0.873	0.491	2.313	-0.669	0.659	0.512	0.310	0.141	1.865
Family caregivers (yes)	-0.156	0.278	0.856	0.575	0.496	1.476	-0.238	0.468	0.788	0.611	0.315	1.972
Social group participation (yes)	0.162	0.293	1.175	0.582	0.661	2.089	-0.535	0.551	0.586	0.332	0.199	1.726
Family social contact (high)	-1.356	1.055	0.258	0.199	0.033	2.039	-0.672	1.320	0.511	0.611	0.038	6.795
Satisfaction of family support	-0.344	0.189	0.709	0.069	0.490	1.027	0.356	0.272	1.428	0.190	0.839	2.432
Satisfaction of friends/Neighbors support	-0.068	0.121	0.934	0.570	0.737	1.183	-0.085	0.190	0.918	0.652	0.633	1.331
Using Internet (yes)	0.031	0.281	1.032	0.912	0.594	1.790	0.217	0.487	1.243	0.656	0.478	3.230
Having pets (yes)	-0.056	0.381	0.946	0.884	0.448	1.997	1.200	0.877	3.319	0.172	0.595	18.531
Physically active leisure (yes)	-0.653	0.276	0.521	0.018	0.303	0.894	-0.676	0.540	0.509	0.211	0.177	1.466
Sedentary leisure (yes)	0.581	0.296	1.787	0.050	1.000	3.194	0.248	0.483	1.282	0.607	0.498	3.301
Change in Loneliness during COVID-19 (increased)	0.409	0.283	1.505	0.149	0.864	2.622	-0.931	0.477	0.394	0.051	0.155	1.003
Worry about COVID-19	0.310	0.147	1.363	0.035	1.023	1.817	-0.369	0.236	0.692	0.118	0.436	1.098
Model fit	-2 log likelihood = 473.516, Chi-square = 235.700 (d.f. = 36), *p* < 0.001						-2 log likelihood = 185.539, Chi-square = 195.709 (d.f. = 36), *p* < 0.001					

Note: Analysis by logistic regression. The reference group: region (north), living area (urban), age (65–74), sex (female), marital status (no spouse), living arrangement (with others), smoking (no), drinking (no), physical activity (less than 150 min per week), work (no), family care (no), social group participation (no), family social contacts (low), using internet (less than once per week), having pet (no), physically active leisure (no), sedentary leisure (no), and change in loneliness during COVID-19 (reduced or no change). Other variables were ordinal or continuous. The constant term was omitted in the table

## Discussion

This cross-sectional study examined the associations of loneliness literacy with loneliness and changes in loneliness during the COVID-19 pandemic among older people in Taiwan. Having higher loneliness literacy was related to lower loneliness, and loneliness literacy was related to different changes in loneliness during COVID-19. Loneliness level and loneliness literacy were related to the risk of depressive symptoms and poor life satisfaction.

### Loneliness literacy and loneliness

The concept of loneliness literacy used in this study was modified from the Loneliness Literacy Scale [13]. The original scale consisted of dimensions of self-efficacy, perceived social support, motivation, subjective norm, and two unused dimensions (i.e., knowledge and attitudinal belief). In this study, four dimensions of loneliness literacy based on factor analysis were found, namely, self-efficacy, social support, in-home support, and socialization. Some items in the original scale were not included because the pretest participants scored them very low, such as formal service knowledge, motivation and subjective norm items. Additionally, support from family and help at home were separated from the original perceived social support factor. The results of factor analysis suggest that as a family-centered culture, a family bond may exert a stronger influence on older people than bonds from other sources, such as friends or neighbors. Older people in Taiwan also expect more from their family than from friends or neighbors. Thus, support from family and help at home were separate from social support from friends or neighbors in this Taiwanese sample.

Higher *self-efficacy* and *in-home support* were related to a lower level of loneliness among community-based older adults in this study. This finding supports the hypothesis of the resource perspective. *Self-efficacy* represents information and resources known and available in the neighborhoods of older people and they have the confidence to use these resources in daily life. *In-home support* means support at home, either from their family or from in-home services. In fact, from the qualitative descriptions of the context of loneliness in the open-ended question, many participants felt lonely when they were sick, disabled, or needed help but were without assistance. *Self-efficacy and in-home support* are modifiable dimensions that can be addressed to reduce the manifestation of loneliness. To facilitate the provision of neighborhood resources, the accessibility of community networks and information should be developed. *In-home support* can be encouraged and strengthened by providing home-based or alternative community-based services.

### Changes in loneliness during COVID-19

During a pandemic event, such as COVID-19, disease control policies, social distancing, and worry about the risk of COVID-19 can increase the risk of feeling lonely [12]. Among the four dimensions of loneliness literacy, *self-efficacy*, *social support, and socialization* were related to changes in loneliness during COVID-19, but in opposite directions. Having more *self-efficacy* reduced the possibility of a change in loneliness (increase) due to COVID-19. It is possible that when older people have more information and knowledge about COVID-19 or have a greater ability to acquire resources from the community, they are less likely to experience loneliness during the pandemic. In contrast, older adults who had high levels of *social support* (i.e., higher social support from friends or neighbors) and higher *socialization* reported an increase in loneliness during the COVID-19 pandemic. One explanation is that these people may be affiliated with people outside their families, but usual social interaction was not allowed during the COVID-19 pandemic, thus contributing to an increase in loneliness.

In addition to loneliness literacy, it was found that being male, engaging in emotion-focused coping strategies, and having lower satisfaction with family support were related to increased loneliness during COVID-19, which is in line with the literature. Females tend to have better psychological resilience and are less likely to feel lonely [28]. Engaging in problem-focused coping, rather than emotion-focused coping strategies, would be more realistic under the uncertainty of a new pandemic disease and, thus, may make older persons more robust against increased loneliness [29]. A satisfactory family relationship could lower loneliness, especially among older males. Interestingly, performing physically active exercise was related to higher odds of reporting feeling lonelier because of COVID-19. It is possible that physical activity can expedite the ability to regulate emotion during the COVID-19 outbreak, and physical and leisure activities may have been prohibited or restricted. Therefore, those who used to perform physical and leisure activities may feel more disruptions in their daily routine and perceive their loneliness level as high.

### Loneliness and mental well-being

Previous studies indicated that loneliness was related to more depressive symptoms [3, 30–32] and poor life satisfaction [4, 17, 33–35]. In accordance with previous studies, high levels of loneliness were significantly associated with having depressive symptoms and poor life satisfaction when other covariates were controlled for.

In the four dimensions of loneliness literacy, high *self-efficacy* was related to high life satisfaction, and high *in-home support* was related to few depressive symptoms. This investigation advances current knowledge by confirming that increased *self-efficacy* and *in-home support* may be helpful to increase mental well-being. Providing more information to increase loneliness-related knowledge and efficacy may help older people access home and community-based care and manage their lives in the community, leading to higher life satisfaction. Additionally, social support and assistance from family members are particularly important for lowering the depressive symptoms of community-dwelling older individuals in a family-centered society, such as Taiwan.

Engaging in more emotion-focused coping strategies was related to more depressive symptoms, which is consistent with previous research [25, 36]. Worry about COVID-19 was also related to higher depressive symptoms [12], implying that educating people on how to adopt active coping strategies in response to stress caused by the pandemic is important in protecting mental health and has the potential to prevent further mental conditions. Additionally, consistent with previous studies, older adults performing physically active and leisure activities are less likely to develop cognitive impairment and depressive symptoms [37, 38], suggesting that physical activity or leisure may increase physical function and protect cognitive function and emotional health.

### Limitations

There were some limitations in this study. First, due to the COVID-19 pandemic, the survey data was not collected through face-to-face interviews; rather telephone interviews were conducted. The interview time had to be shortened, and some of the questions could not be included in this survey, such as the resilience scale and details of health conditions. In addition, some questions about loneliness were not easy to explore, such as the open-ended questions regarding the context of loneliness and perceived consequences. Second, as a cross-sectional study, the causal relationship of the variables cannot be confirmed. Third, the sample was drawn by the Computer-Assisted Survey Telephone Interview System, and the sampling was based on the population distribution of the region, not by cities. Thus the sample may not be generalizable to cities, and the sample of older people may not be generalizable to the entire population.

## Conclusion

In conclusion, higher *self-efficacy* of loneliness literacy, engaging in problem-focused coping rather than emotion-focused coping strategies, and having higher levels of satisfaction with family support were related to a lower chance of increased loneliness during the pandemic and were also protective factors for mental well-being in older adults. Building up an age-friendly community to provide home- and community-based services is suggested. Providing accessible information and enabling social connectedness in the community for older adults should be actions taken by the government. Learning positive (problem-focused) coping strategies and building up resilience under stress are highly suggested in health education to promote mental health. A longitudinal survey should be conducted for following up on the mental health of older adults during the pandemic and to explore the effects of geographical resources on loneliness in future research. An intervention study is also suggested to examine whether the provision of community-based services and an age-friendly social network may reduce loneliness.

## Supplementary Information

**Additional file 1: Table S1.** Descriptive analysis of the 6-item Revised UCLA Loneliness Scale. **Table S2.** Factor analysis of loneliness literacy scale.

## Data Availability

The datasets used and/or analysed during the current study are available from the corresponding author on reasonable request.
