# Supplementary material for: Loneliness, loneliness literacy, and change in loneliness during the COVID-19 pandemic among older adults: a cross-sectional study
Source: BMC Geriatr. 2022 Aug 26;22:707. doi: 10.1186/s12877-022-03396-7 (PMC9412798; doi:10.1186/s12877-022-03396-7)
Supplement: Supplementary file 1 — Additional file 1: Table S1. Descriptive analysis of the 6-item Revised UCLA Loneliness Scale. Table S2. Factor analysis of loneliness literacy scale. [file 12877_2022_3396_MOESM1_ESM.docx]

**Supplementary Material**

Table S1. Descriptive analysis of the 6-item Revised UCLA Loneliness Scale

| Questions (How often do you feel …) | Mean | S.D. |
| --- | --- | --- |
| You lack companionship? | 1.34 | 0.800 |
| You feel alone? | 1.51 | 0.996 |
| You are no longer close to anyone? | 1.29 | 0.748 |
| You feel left out? | 1.20 | 0.618 |
| You feel that no one really knows you well? | 1.36 | 0.792 |
| You feel that people are around you but not with you? | 1.34 | 0.787 |
| Total score of loneliness | 8.01 | 3.280 |

Table S2. Factor analysis of loneliness literacy scale

| Items | *Self-Efficacy* | *Social Support* | *Socialization* | *In-home Support* |
| --- | --- | --- | --- | --- |
| Do most things you truly want to do | **.778** | .044 | .065 | -.066 |
| Attend activities alone | **.774** | -.014 | .121 | -.131 |
| Aware of information (exercise programs, entertainment, or courses) in daily life | **.681** | .037 | .058 | .162 |
| Nothing can stop you to participate in an activity | **.672** | -.018 | .291 | -.028 |
| Arrange to find someone to help if you need | **.559** | -.078 | .101 | .130 |
| Know how to attend activities in the community | **.511** | -.090 | .330 | .241 |
| Arrange transportation services | **.504** | .021 | .175 | .329 |
| Know the information of exercise, entertainment or courses | **.467** | .014 | .307 | .223 |
| Neighbors would help if you ask | -.013 | **.997** | -.030 | -.007 |
| Friends would help if you ask | -.014 | **.997** | -.034 | -.011 |
| Feel important to remain among the people | .104 | -.030 | **.787** | .003 |
| Take initiative to introduce yourself | .122 | -.003 | **.711** | .005 |
| Speak up regularly in a group of acquaintances | .329 | -.035 | **.506** | .036 |
| Family would help you if you ask | -.068 | -.011 | -.142 | **.777** |
| Arrange in-home services | .296 | -.009 | .239 | **.536** |
| Note. N=772. Extracted by Principal Component Analysis with Varimax rotation; explained variance=55.94%. | | | | |
